# TMEM97 governs partial epithelial-mesenchymal transition of retinal pigment epithelial cells via the CTNND2-ADAM10 axis

**DOI:** 10.1016/j.omtn.2025.102460

**Published:** 2025-01-21

**Authors:** Jing Li, Yosuke Nagasaka, Hongtao Shen, Xinyu Zhou, Jianjie Ma, Dilza Trevisan-Silva, Nicholas E. Sherman, Jayakrishna Ambati, Bradley D. Gelfand, Lian-Wang Guo

**Affiliations:** 1 Division of Surgical Sciences, Department of Surgery, School of Medicine, University of Virginia, Charlottesville, VA 22903, USA; 2 Department of Ophthalmology, University of Virginia, Charlottesville, VA 22903, USA; 3 School of Medicine Core Facilities, University of Virginia, Charlottesville, VA 22903, USA; 4 Department of Molecular Physiology and Biological Physics, University of Virginia, Charlottesville, VA 22903, USA

**Keywords:** MT: Oligonucleotides: Therapies and Applications, partial EMT, E-cadherin, N-cadherin, ZO-1, catenin, sigma-2 receptor, RPE, AMD, retinal degeneration, photoreceptor

## Abstract

Epithelial-mesenchymal transition (EMT) is associated with retinal pigment epithelium (RPE) dysfunction in degenerative retinal diseases. However, the role of partial EMT (pEMT), a hybrid state exhibiting both epithelial and mesenchymal markers, remains poorly understood in this context. Our previous research demonstrated that TMEM97 ablation in mice worsens photoreceptor loss in an oxidant-induced RPE damage model. Here, we link TMEM97 to pEMT in RPE cells and explore the underlying molecular mechanisms. We found that re-expressing TMEM97 in the RPE of TMEM97-knockout mice, via subretinal lentiviral delivery, mitigated oxidant (NaIO_3_)-induced photoreceptor loss. Interestingly, TMEM97 knockout in ARPE19 cells *in vitro* led to upregulation of cadherin/adhesion-binding pathways, even without oxidant exposure. Integrated proteomic, transcriptomic, segmentation, and immunoblot analyses revealed that TMEM97 ablation induces pEMT, marked by the concurrent expression of epithelial E-cadherin and mesenchymal N-cadherin, a process reversed upon TMEM97 re-expression. Furthermore, TMEM97 negatively regulated CTNND2 protein (catenin δ-2), but not the known EMT driver β-catenin, and CTNND2 was found to promote ADAM10, which sustains both E- and N-cadherin protein levels. These findings identify TMEM97 as a novel regulator of RPE-cell pEMT through the CTNND2-ADAM10 axis, highlighting potential new targets for therapeutic intervention in RPE-related pathophysiology.

## Graphical abstract

**Figure:**
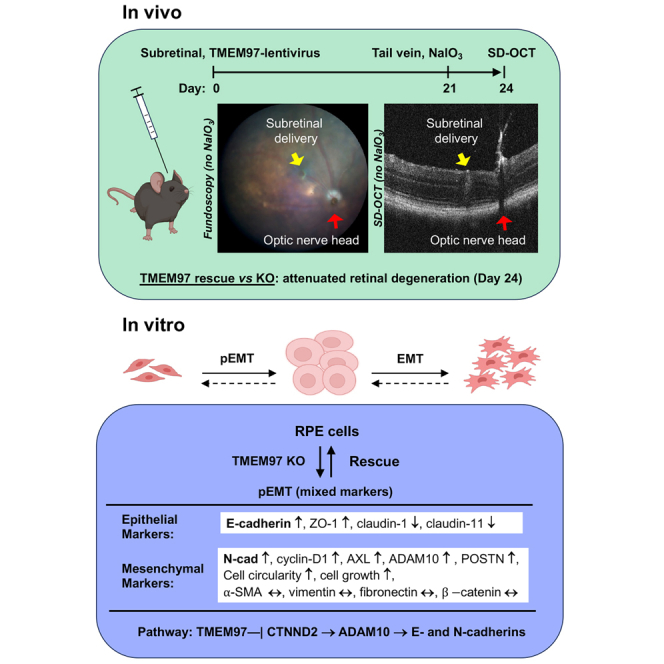

## Teaser

The epithelial-mesenchymal transition (EMT) is associated with retinal pigment epithelium (RPE) dysfunction/disease. However, partial EMT (pEMT), a hybrid state, remains little understood in RPE cells. The study by Li and colleagues, the first to specifically focus on RPE-cell pEMT, identifies TMEM97 as a novel regulator acting via the CTNND2-ADAM10 axis.

## Introduction

The retinal pigment epithelial (RPE) cells constitute a monolayer between the neural retina and the choroid, crucial for maintaining photoreceptors and normal vision. However, their unique location makes them vulnerable to various insults, including oxidative stress, inflammation, and harmful molecules from the choroid and photoreceptor outer segments. Dysfunction of RPE cells plays a pivotal role in the pathogenesis of retinal diseases,^1^ underscoring the need to discover underlying mechanisms for developing effective therapies.

One significant manifestation of RPE dysfunction is the epithelial-mesenchymal transition (EMT), implicated in human retinal diseases like age-related macular degeneration (AMD) and proliferative vitreoretinopathy.^2^ EMT is classically defined as the complete loss of epithelial traits and gain of a mesenchymal phenotype. Recent advances in cell fate mapping technologies, however, led to the discovery of partial EMT (pEMT), whereby cells exhibit hybrid states expressing both epithelial (e.g., E-cadherin) and mesenchymal (e.g., N-cadherin) markers.^3^^,^^4^ pEMT is linked to more aggressive tumor behavior than complete EMT.^5^ Despite its clinical significance, the mechanisms governing pEMT remain largely obscure. Moreover, pEMT in RPE cells is little studied,^6^^,^^7^^,^^8^ necessitating research to identify potential therapeutic targets.

Recent meta-analyses of genome/transcriptome-wide association studies have identified transmembrane protein 97 (TMEM97) as a novel risk locus for AMD,^9^^,^^10^ although functional verification is pending. Studies, including ours, indicated that TMEM97 is involved in RPE cell stress responses and retinal degeneration.^11^^,^^12^^,^^13^^,^^14^ Primarily studied in cancers, TMEM97 is also linked to cholesterol homeostasis, Niemann-Pick disease, Alzheimer disease, and neuropathic pain.^14^^,^^15^^,^^16^^,^^17^^,^^18^^,^^19^ Intriguingly, *TMEM97* was recently identified as the long sought-after coding gene of the sigma-2 receptor (S2R).^20^ An array of experimental and pharmaceutical S2R-targeting compounds has been developed for treating cancers and psychiatric disorders.^21^ However, the S2R/TMEM97 gene-specific molecular functions remain elusive,^13^^,^^22^ limiting their potential as a therapeutic target for clinical translation.

Building upon our previous work using TMEM97-knockout (KO) mice and the oxidant-induced RPE-damage model,^11^ here, we further investigated the specific role of TMEM97 in the retina. We re-expressed TMEM97 locally in the RPE of *Tmem97*^*−/−*^ mice and observed an amelioration of retinal degeneration. We then performed *in vitro* analyses of proteomics, transcriptomics, and morphological segmentation using wild-type (WT) and TMEM97 KO human ARPE19 cells. The study led to an unexpected finding: independent of oxidant treatment, TMEM97 deletion induced a pEMT phenotype with simultaneous upregulation of E- and N-cadherin proteins. Mechanistically, we identified a novel TMEM97-CTNND2-ADAM10 pathway regulating pEMT. These findings may advance the understanding of RPE cell biology, informing potential new interventional strategies for treating retinal diseases.

## Results

### Rescue of TMEM97 expression in the *Tmem97*^*−/−*^ mouse retina ameliorates oxidant-induced photoreceptor loss

**Figure 1:**
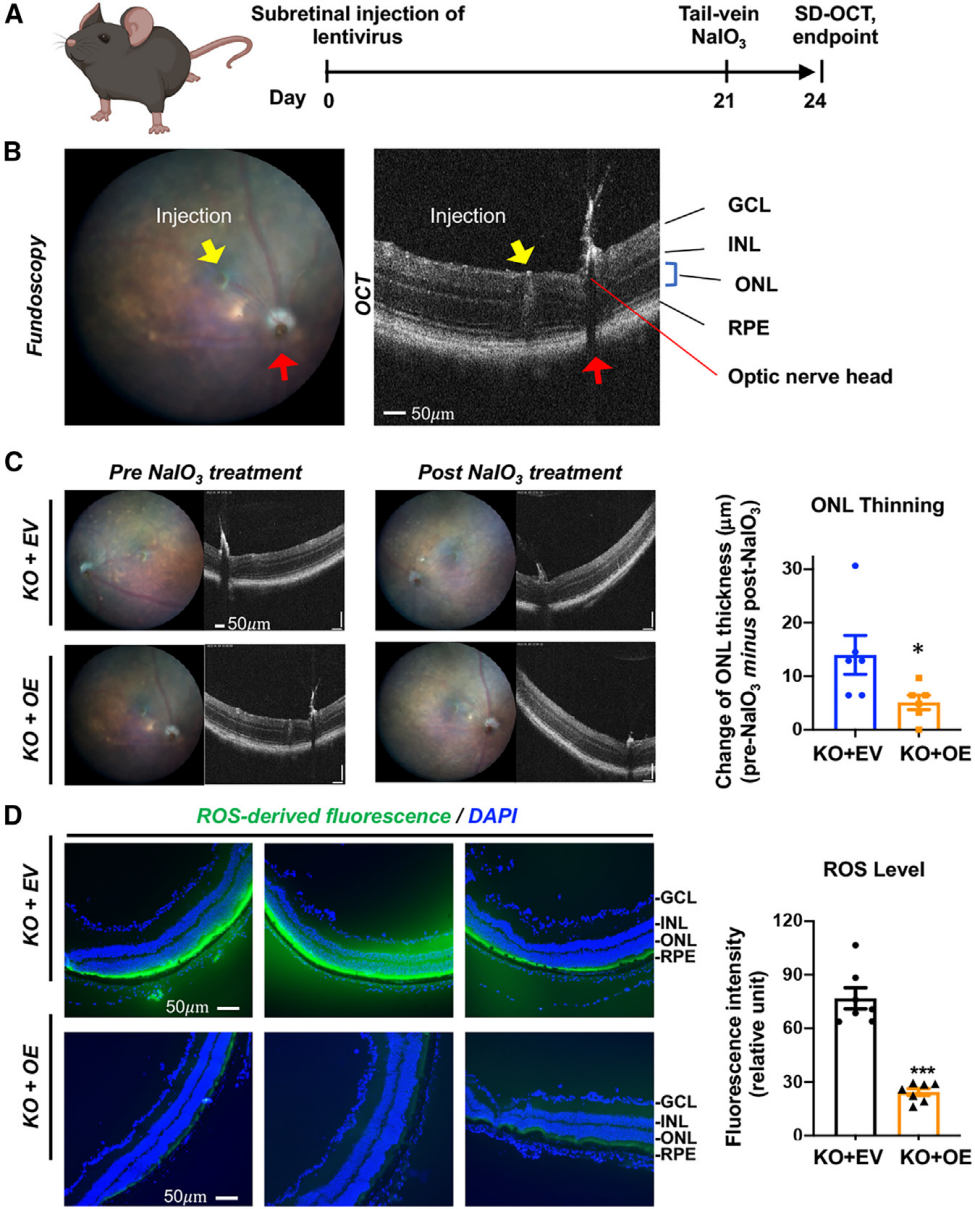
Rescue of TMEM97 expression in the retina of *Tmem97*^*−/−*^ mice mitigates oxidant-induced photoreceptor loss Lentivirus for an empty vector (EV, GFP only) or TMEM97 rescue (TMEM97-GFP) was subretinally delivered into male TMEM97 KO mice and allowed to express for 3 weeks. The mice then received a single tail-vein injection of NaIO_3_ (30 mg/kg). SD-OCT was recorded 1 day before and 3 days after NaIO_3_ injection, and the decrease in ONL thickness (called here ONL thinning) was measured in the region proximal to the subretinal injection site. The mice were then euthanized for retinal cryosection preparation and ROS detection. Quantification: fluorescence intensity values from 3 to 4 sections were averaged for each animal, and the averages from all animals in each group were averaged again to produce a mean ± SEM. GCL, ganglion cell layer. INL, inner nuclear layer. ONL, outer nuclear layer. RPE, retinal pigment epithelium. (A) Schematic illustrating the experimental timeline. (B) Enlarged fundus image and SD-OCT image (pre-NaIO_3_) showing the site of subretinal injection of lentivirus (yellow arrow) and the site of the optic nerve head (red arrow). Scale bar: 50 μm. (C). ONL thinning. Representative fundus images and SD-OCT images show positions of subretinal injection and ONL thicknesses, respectively. Quantification: mean ± SEM, *n* = 6 male mice; Student’s t test: ∗*p* < 0.05. Scale bar: 50 μm*.* (D) ROS levels. ROS was detected in retinal cryosections stained with H2DCFDA (3 days post-NaIO_3_). Shown are representative images from 3 mice in each group. Quantification: mean ± SEM, *n* = 6 male mice; Student’s t test: ∗∗∗*p* < 0.001. Scale bar: 50 μm.

In our previous study using the NaIO_3_-induced RPE damage model, we observed exacerbated retinal degeneration in *Tmem97*^*−/−*^ mice compared to *Tmem97*^*+/+*^ controls.^11^ It is important to determine whether this effect was specifically attributable to TMEM97, considering possible compensatory responses in the systemic TMEM97 KO mice. To address this, here, we performed a rescue experiment by re-expressing TMEM97 in the KO background (Figures 1A and 1B). The lentivirus for overexpression (OE) of TMEM97-GFP or the empty vector (EV) control was injected into the subretinal space of *Tmem97*^*−/−*^ mice and allowed to express for 3 weeks. Retinal degeneration was then induced via tail-vein injection of NaIO_3_ (Figure 1A). We confirmed the expression of TMEM97-GFP in ARPE19 cells (Figure S1A) and also in RPE whole mounts (Figure S1B). Optical coherence tomography (OCT) analysis revealed that photoreceptor loss, indicated by the thinning of the outer nuclear layer (ONL), was attenuated in the TMEM97-rescue group compared to the control group 3 days after NaIO_3_ injection (Figure 1C). In addition, the reactive oxygen species (ROS) signal was lower in TMEM97-rescue retinas compared to EV controls (Figure 1D). These data support a TMEM97-specific function in countering ROS elevation and mitigating photoreceptor loss in the oxidant-induced RPE-damage model.

**Figure 2:**
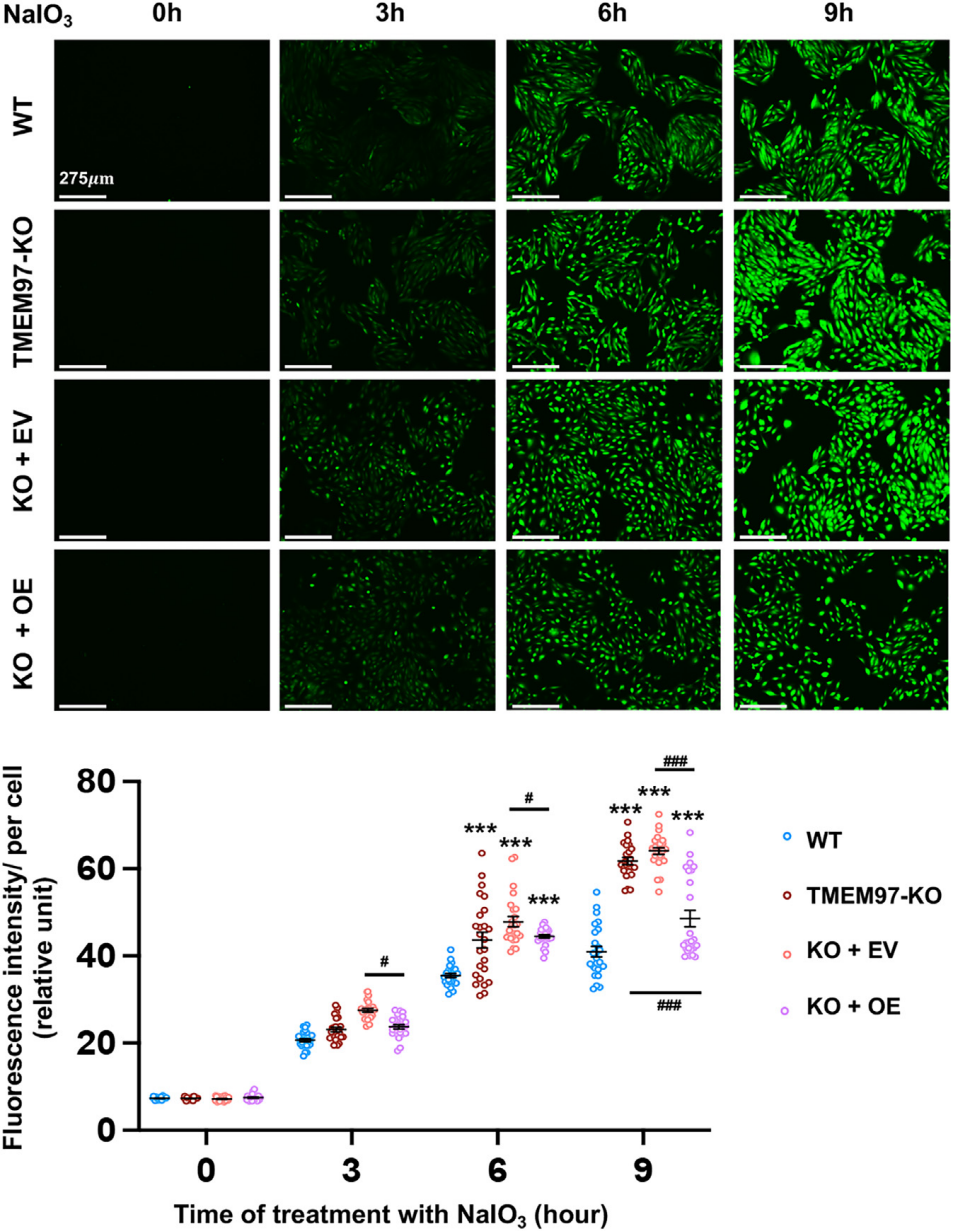
Rescue of TMEM97 expression in TMEM97 KO ARPE19 cells hampers oxidant-induced ROS elevation ARPE19 cell culture and ROS detection are described in materials and methods. Three independent cell cultures (*n* = 3) were used, and 25 slides were imaged and averaged to generate the mean ± SEM. Statistics: two-way ANOVA; ∗∗∗*p* < 0.001 (compared to the respective WT control); ^#^*p* < 0.05; ^###^*p* < 0.001 (pairwise comparison).

With the effect of TMEM97 rescue on ROS attenuation observed *in vivo*, we further conducted *in vitro* TMEM97 rescue experiments using both WT (*TMEM97*^*+/+*^) and KO (*TMEM97*^*−/−*^) human ARPE19 cell lines that we generated via CRISPR genome editing.^11^ For oxidative challenge, cells were treated with 5 mM NaIO_3_, a condition utilized in our previous report.^11^ As shown in Figure 2, treatment with NaIO_3_ resulted in higher levels of ROS in KO cells compared to WT cells at 6 and 9 h. While transduction of KO cells with the EV did not alter ROS levels (KO + EV vs. KO), TMEM97 rescue in KO cells reduced ROS throughout 3–9 h of the NaIO_3_ treatment (KO + OE vs. KO + EV). These assays indicate that the rescue of TMEM97 expression in the KO background suppresses ROS production. Taken together, the *in vivo* and *in vitro* results indicate a TMEM97-specific function in moderating ROS under the NaIO_3_ treatment.

### Proteomics indicates an upregulation of cadherin/adhesion-binding pathways in *TMEM97*^*−/−*^ ARPE19 cells irrespective of oxidant treatment

**Figure 3:**
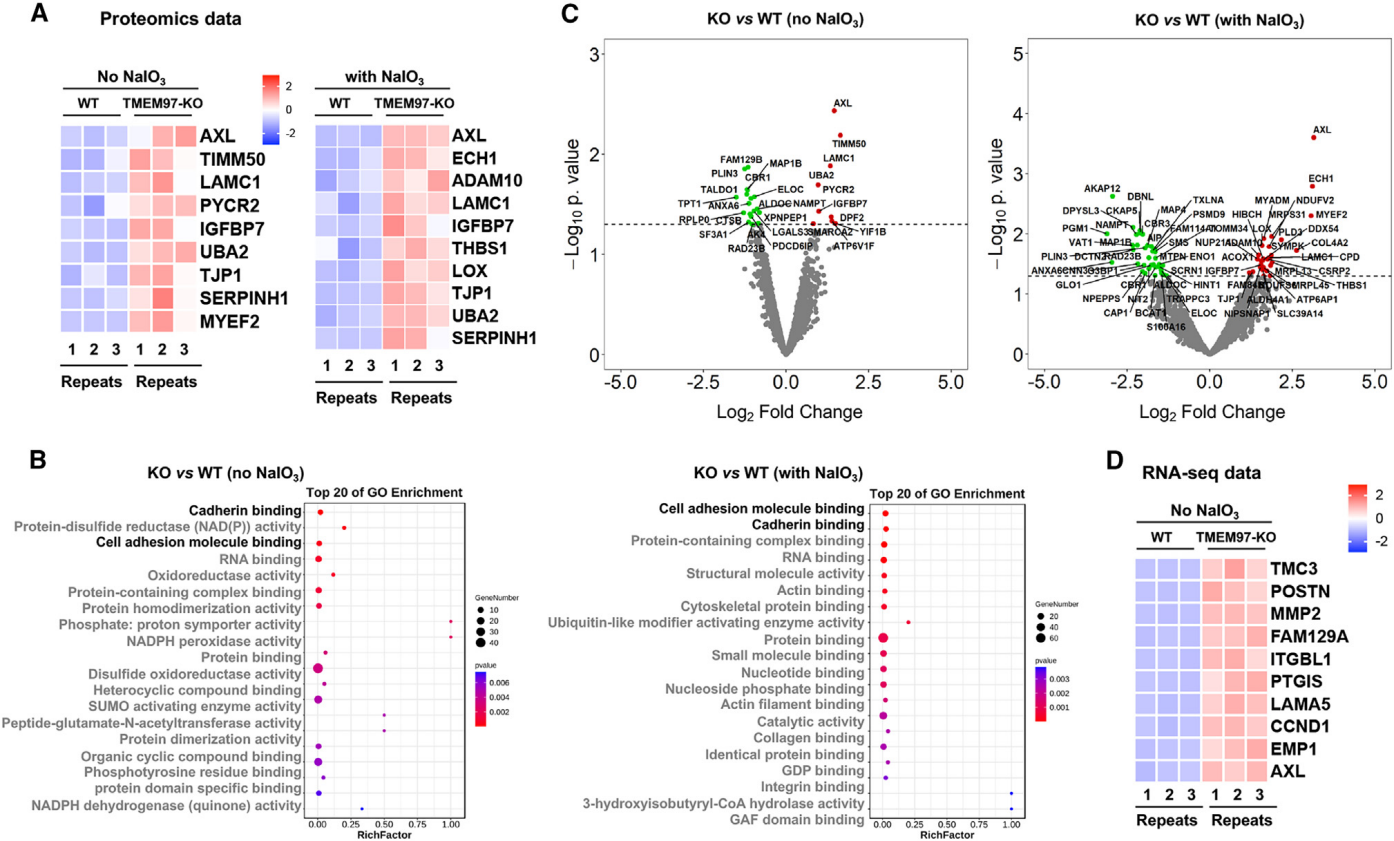
Proteomics analysis of the impact of TMEM97 ablation on protein expression in ARPE19 cells *TMEM97*^*+/+*^ (WT) and *TMEM97*^*−/−*^ (KO) ARPE19 cells were cultured to full confluence in the regular growth medium. For proteomic analysis, 4 conditions were applied: WT and KO cells were treated without or with 5 mM NaIO_3_ for 24 h before harvest for mass spectrometry. For transcriptomic analysis, WT and KO cells were cultured without NaIO_3_. Three independent cultures (*n* = 3) of WT or KO cells were used. (A) Proteomics heatmap. Top 10 best *q* values (*p*adj, adjusted *p* value) for upregulated proteins (KO vs. WT) were selected, and the proteins were ranked by their fold changes; *n* = 3. (B) GO enrichment. Presented are the top 20 upregulated pathways ranked by best *q* values (*p*adj). (C) Volcano plots. Red and green dots represent up- and downregulated proteins, respectively. The dashed line is a threshold of *p* = 0.05. (D) Transcriptomics heatmap. Top 10 best *q* values (*p*adj) for upregulated genes (KO vs. WT) were selected, and the genes were ranked by their expression fold changes; *n* = 3.

While an involvement of TMEM97 in RPE physiology and associated retinal degeneration has been implicated in our and others’ reports,^11^^,^^12^^,^^23^ the molecular/cellular function of TMEM97 remains poorly understood. We therefore next sought to uncover the TMEM97 molecular pathways via proteomics using WT and KO ARPE19 cells with or without NaIO_3_ treatment (Figure 3A). We focused on upregulated proteins (KO vs. WT) because we had already analyzed downregulated genes in our recent transcriptomic study.^23^ Interestingly, Gene Ontology (GO) pathway analysis revealed cadherin binding and adhesion molecule binding as top-ranked upregulated pathways (Figure 3B). Heatmaps further illustrated that AXL receptor tyrosine kinase (AXL) and a-disintegrin-and-metalloprotease (ADAM10), both involved in cadherin binding,^24^ were top-ranked (Figure 3A), consistent with volcano plots (Figure 3C). Other junction/adhesion proteins also appeared on the top-rank lists, including tight junction protein 1 (TJP1, also known as ZO-1, or zonula occludens-1), PYCR1, LAMC1, LOX, and THBS1 (Figure 3A).

Of note, the TMEM97 regulation of cadherin/adhesion-binding pathways occurred regardless of oxidant treatment (Figure 3B), which was unexpected, as we initially planned to investigate redox responses based on the results presented in Figure 2. Accordingly, in the following analyses, we narrowed down our experimental setting to cell culture conditions without NaIO_3_ (e.g., the transcriptomic analysis, Figure 3D).

**Figure 4:**
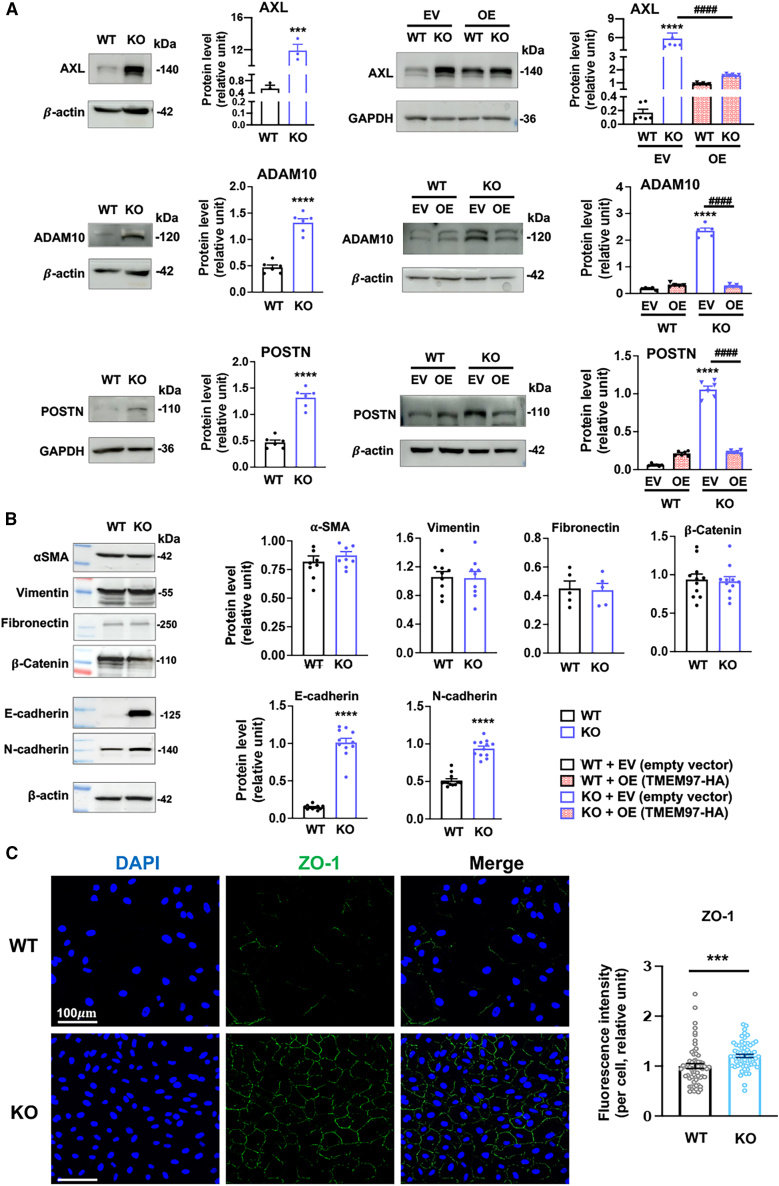
Immunoblots indicate that TMEM97 suppresses E-cadherin and N-cadherin protein expression To align WT and TMEM97 KO ARPE19 cells to an epithelial cell state, the cells were cultured to full confluence and then maintained for 3 days prior to harvest for immunoblot assays. For the rescue of TMEM97 expression, lentivirus for the EV control or for TMEM97 OE was used to transduce WT and KO cells that reached full confluence and incubated for 3 days prior to harvest for immunoblotting. Quantification: mean ± SEM, *n* = 3–8 independent repeat experiments (indicated by data points in A and B). Student’s t test (2 groups) or one-way ANOVA/Tukey (4 groups): ∗∗*p* < 0.01; ∗∗∗*p* < 0.001; ∗∗∗∗*p* < 0.0001 (compared to the first bar); ^####^*p* < 0.0001 (pairwise comparison). (A) TMEM97 negates the protein levels of AXL, ADAM10, and POSTN. (B) TMEM97 ablation has no effect on α-SMA, vimentin, fibronectin, and β-catenin protein levels but does result in upregulation of E-cadherin and N-cadherin proteins. (C) TMEM97 ablation increases the ZO-1 protein level. For the immunofluorescence assay, ARPE19 cells were cultured to subconfluence. Quantification: For WT or KO cells, 64 images were taken, and the fluorescence intensities of 16 areas within each image were measured using ImageJ and averaged; the mean ± SEM of each group was then calculated. Statistics: Student’s t test; ∗∗∗*p* < 0.001.

We next focused on the cadherin-associated proteins AXL and ADAM10, given their prominent upregulation based on the proteomics data (Figure 3A). Immunoblots indicated an approximately 20-fold increase of AXL in TMEM97 KO cells compared to WT cells (Figure 4A). Furthermore, rescue (for TMEM97 blot, see Figure S2) of TMEM97 expression in KO cells reduced the AXL protein level that was elevated due to TMEM97 ablation. Similarly, ADAM10 protein was upregulated in KO cells, and TMEM97 rescue abolished this upregulation (Figure 4A). Consistent with the proteomics data (heatmaps in Figure 3A), the TMEM97 KO-induced upregulation of AXL and ADAM10 was also observed in the presence of NaIO_3_ (Figure S3).

In addition, periostin (POSTN), a well-known junction-associated matricellular protein,^25^ was top-ranked on the transcriptomics heatmap (Figure 3D) and exhibited a protein expression pattern similar to that of AXL and ADAM10 on immunoblots (Figure 4A). Therefore, proteomics and transcriptomics along with immunoblot analysis demonstrate that TMEM97 negatively regulates the expression of AXL, ADAM10, and POSTN in ARPE19 cells.

### TMEM97 ablation in ARPE19 cells induces a pEMT phenotype with concurrent E- and N-cadherin expression

Of note, AXL, ADAM10, and POSTN are known regulators of EMT.^24^^,^^26^ Moreover, multiple other top-ranked upregulated proteins or genes are involved in EMT, for example, LOX,^27^ LAMC1,^28^ SERPINH1,^29^ IGFBP7,^30^ and ZO-1^31^ on the proteomics heatmap (Figure 3A), and ITGBL1,^32^ PTGIS,^33^ LAMA5,^34^ MMP2,^35^ FAM129A,^35^ and EMP1^36^ on the transcriptomics heatmap (Figure 3D). This led us to hypothesize that TMEM97 ablation in ARPE19 cells promotes EMT. Surprisingly, we found that some of the classic mesenchymal markers such as α-smooth muscle actin (α-SMA), vimentin, fibronectin, and β-catenin remained unchanged in TMEM97 KO cells compared to WT cells (Figure 4B).

In the literature, EMT is often synonymous with the E-cadherin to N-cadherin switching.^37^^,^^38^ We thus determined their protein levels. Interestingly, while the mesenchymal marker N-cadherin was upregulated in KO cells compared to WT cells, the bona fide epithelial marker E-cadherin was also markedly upregulated (Figure 4B), and this upregulation became even more prominent with extended culture time (Figure S4). In addition, immunofluorescence using subconfluent cells indicated that ZO-1 (encoded by the *TJP1* gene), a tight-junction protein and a classic epithelial marker, was upregulated in KO cells relative to WT cells (Figure 4C), validating the proteomics data (Figure 3A).

**Figure 5:**
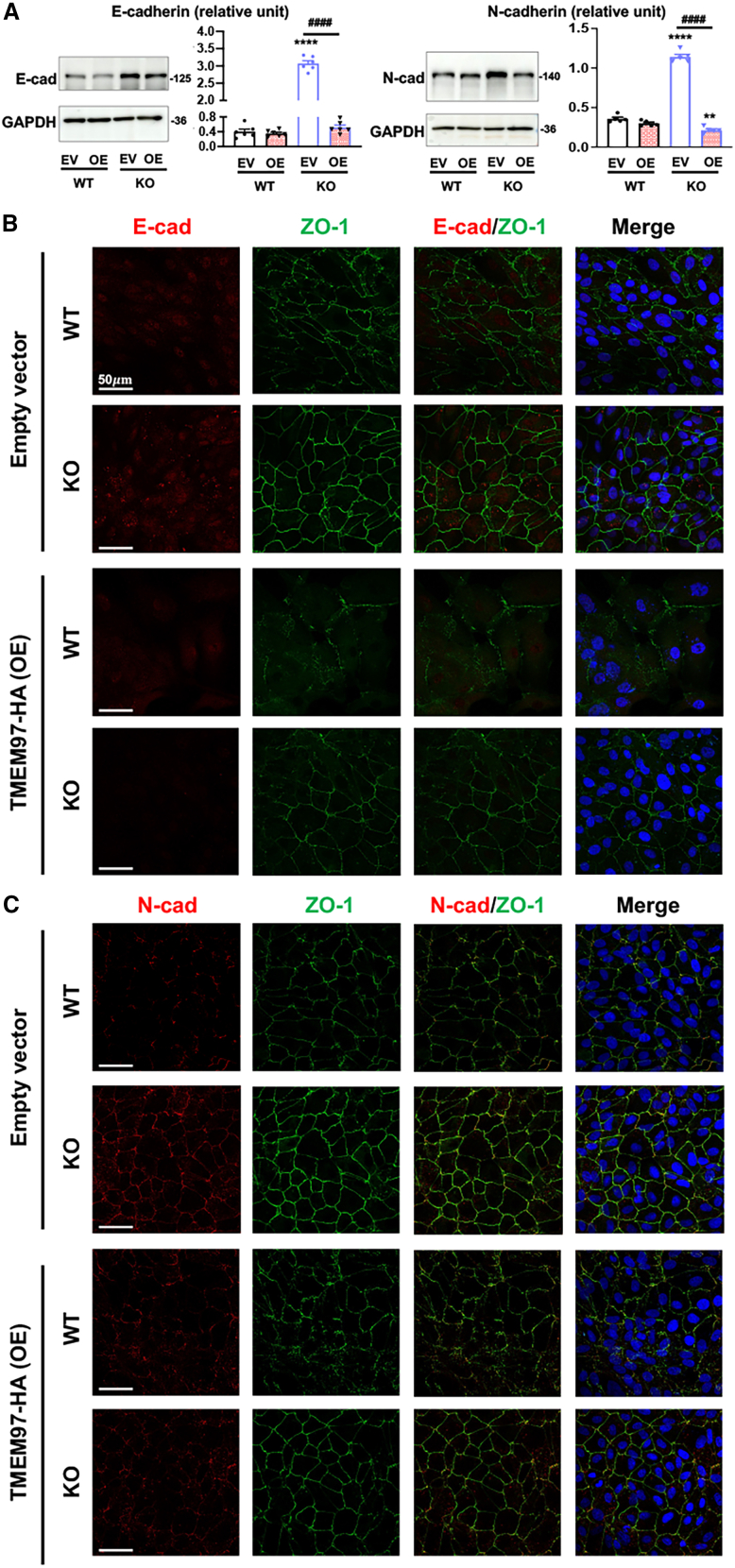
Immunofluorescence indicates upregulation of E- and N-cadherins in TMEM97 KO ARPE19 cells To align WT and TMEM97 KO ARPE19 cells to an epithelial cell state, the cells were cultured to full confluence and then maintained for 3 days prior to assays. For the rescue of TMEM97 expression, WT and KO cells that reached full confluence were added with the lentivirus for the EV control or for TMEM97 OE and cultured for 3 days prior to harvest for immunoblot or immunofluorescence. (A) Immunoblots of E-cadherin and N-cadherin. Quantification: mean ± SEM, *n* = 3–4 independent repeat experiments. Statistics: one-way ANOVA/Tukey; ∗∗*p* < 0.01; ∗∗∗∗*p* < 0.0001 (compared to the first bar, EV/WT); ^####^*p* < 0.0001 (pairwise comparison)*.* (B) Immunofluorescence of E-cadherin (red) and ZO-1 (green). (C) Immunofluorescence of N-cadherin (red) and ZO-1 (green).

To further characterize the TMEM97 regulation of E- and N-cadherin protein levels, we cultured cells to 100% confluence and performed TMEM97 re-expression (rescue) in KO cells. The rescue abolished the KO-induced upregulation of both cadherins, demonstrating a specific role for TMEM97 in negatively regulating these two proteins (Figure 5A). The abolishment of KO-induced upregulation due to TMEM97 rescue was also evident with the immunofluorescence of E- and N-cadherins as well as ZO-1 in overconfluent ARPE19 cells (Figures 5B and 3 days after full confluence). Together, these data obtained with different techniques led to an interesting finding that E-cadherin and N-cadherin, which mark epithelial and mesenchymal states, respectively, are both negatively regulated by TMEM97.

**Figure 6:**
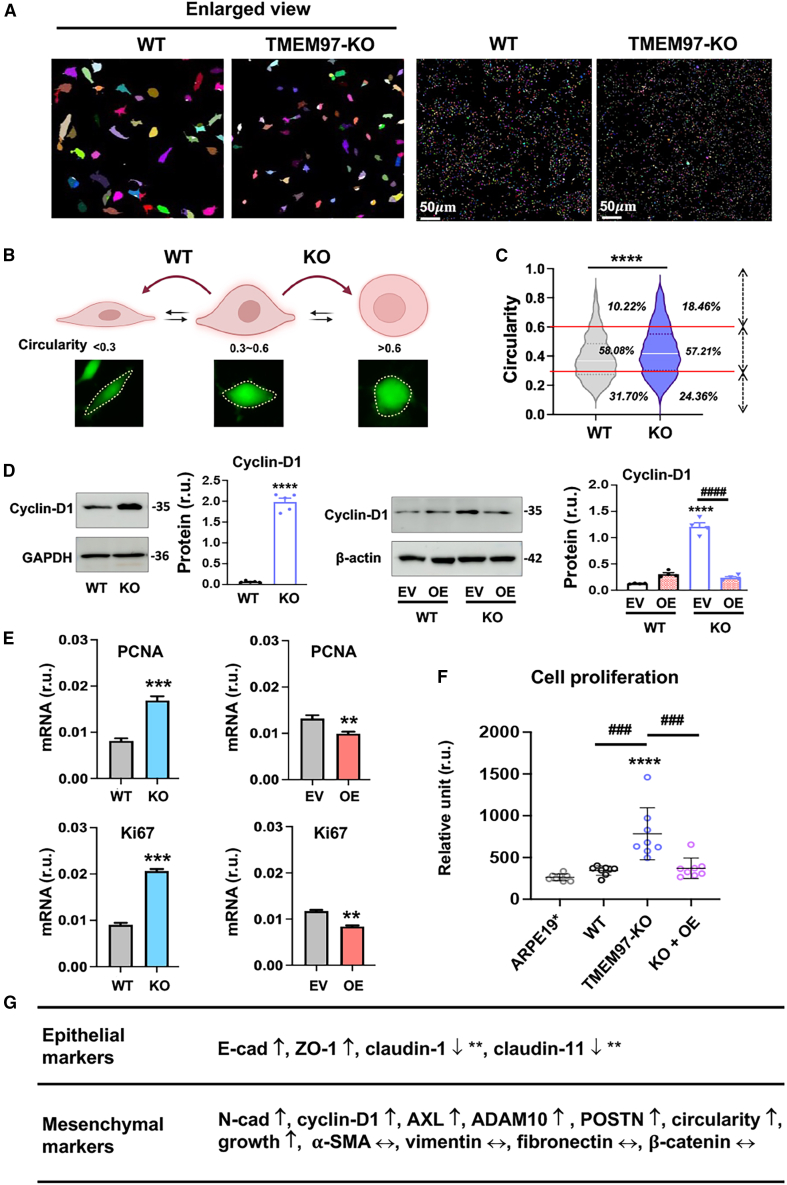
TMEM97 ablation induces ARPE19 cell morphologic change and proliferation To capture changes in cell growth and morphology, WT and TMEM97 KO ARPE19 cells cultured to ∼50% confluence were used for various analyses. For the rescue of TMEM97 expression, lentivirus for the EV control or for TMEM97 OE was used to transduce WT and KO cells for 3 days before harvest for assays. (A) Representative images of cell morphometric segmentation. (B) Schematic of 3 categories of cell circularity. Green images are representative calcein-stained ARPE19 cells. (C) Bin plot showing quantification of cell circularity. Percentages versus total cell numbers are provided for each category. Statistics: Student’s t test; ∗∗∗∗*p* < 0.0001. (D) Immunoblots. Quantification: mean ± SEM, *n* = 3–4 independent repeat experiments. Student’s t test (2 groups) or one-way ANOVA/Tukey (4 groups); ∗∗∗∗*p* < 0.0001 (compared to the first bar); ^####^*p* < 0.0001 (pairwise comparison). (E) mRNA levels determined by qRT-PCR. In this experiment, OE was conducted using a single-clone cell line generated through lentiviral transduction of ARPE19 cells. Quantification: mean ± SD, *n* = 3 repeats. Student’s t test: ∗∗*p* < 0.01; ∗∗∗*p* < 0.001. (F) Cell growth. Cells were stained with the fluorescent dye calcein, and the cell numbers were quantified (each normalized with the cell number at the beginning of the culture). ARPE19∗, the ARPE19 cells without lentiviral transduction. Quantification: Mean ± SD, *n* = 8. One-way ANOVA/Tukey: ∗∗∗∗*p* < 0.0001 (compared to ARPE19∗); ###*p* < 0.001 (pair-wise comparison). (G) Summary of TMEM97 KO-induced changes in epithelial and mesenchymal marker expression. ∗∗Claudin levels based on the transcriptomics data. ↑, upregulated; ↓, downregulated; ↔, no change.

To comprehensively characterize the TMEM97 KO-induced ARPE19 cell phenotypic changes, we used a morphometry segmentation approach to quantify cell circularity (or roundness), an indicator of loss of epithelial cell state.^3^ The circularity of cultured cells was categorized into three levels: high, >0.6; medium, 0.3–0.6; low, <0.3. The analysis indicated that due to TMEM97 ablation, cells with high circularity increased from 10.2% to 18.5% and cells with low circularity decreased from 31.7% to 24.4% (Figures 6A–6C). The proportion of medium-circularity cells was not changed. We next monitored protein levels of CCND1 (cyclin D1), a bona fide pro-proliferative factor and mesenchymal marker. A robust KO-vs.-WT upregulation of cyclin D1 was observed on immunoblots, consistent with the transcriptomics data (Figure 3D).^23^ This upregulation was abolished by the rescue of TMEM97 expression in the KO background (Figure 6D). Other two pro-proliferative factors, PCNA and Ki67, were both upregulated in KO cells, as indicated by their mRNA levels (Figure 6E). In accordance, TMEM97 ablation led to enhanced ARPE19 cell growth, a change reversible upon TMEM97 re-expression in KO cells (Figure 6F). This is consistent with Figure 4C, where an increase in nuclei could be seen in the KO cell culture compared to the WT culture, when the cells were cultured to subconfluence.

As summarized in Figure 6G, TMEM97 ablation resulted in a mix of changes in epithelial and mesenchymal markers. For example, whereas ZO-1 (epithelial) and cyclin D1 (mesenchymal) were both increased, the levels of mesenchymal markers α-SMA, vimentin, fibronectin, and β-catenin did not change. Of particular interest, the classic epithelial marker E-cadherin and mesenchymal marker N-cadherin were both upregulated. These results, along with increased cell circularity and proliferation, collectively indicate that TMEM97 ablation transforms ARPE19 cells into a pEMT state, rather than a complete EMT phenotype.

**Figure 7:**
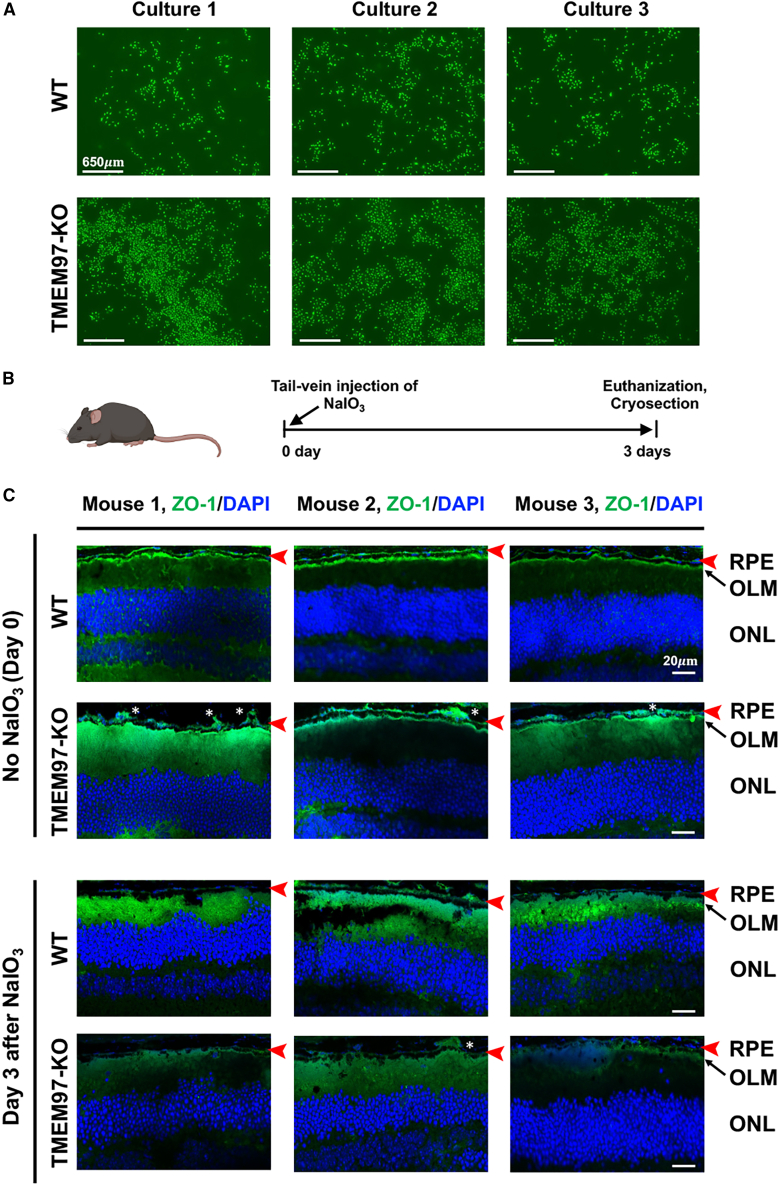
TMEM97 ablation induces ARPE19 cell clustering (A) Clustering of cultured ARPE19 cells. To observe clustering, WT and TMEM97 KO cells were cultured to ∼50% confluence and calcein stained. Three representative images are presented for WT or KO cells. (B) Schematic indicating the mouse model of oxidant-induced RPE damage. Mice received a single tail-vein injection of NaIO_3_ (30 mg/kg) and were euthanized 3 days later. (C) Immunofluorescence of ZO-1 on retinal cryosections. For each animal group, the images were randomly picked from that of 3 mice. Day 0 represents the control group injected with PBS. OLM, outer limiting membrane. Red arrow indicates the RPE layer; ∗ clustering-like RPE cell aggregates. Scale bar: 20 μm.

In addition, it has been reported that a characteristic behavior of cancer cells undergoing pEMT is cell clustering, which enables collective/cluster migration and aggressive tumor progression.^5^^,^^39^ Interestingly, TMEM97 KO ARPE19 cells exhibited a trend of clustering before reaching full confluence (Figure 7A). Moreover, immunofluorescence of ZO-1 illustrated a continuous monolayer of RPE cells in *Tmem97*^*+/+*^ mice, but in *Tmem97*^*−/−*^ mice, a discontinuous layer of RPE cells with cell aggregates was observed (Figures 7B and 7C). The composition and physiology of the cell aggregates observed *in vivo* are not clear at present, but the aggregates seem to be consistent with RPE cell clusters. Therefore, multiple lines of evidence indicate that TMEM97 regulates pEMT in ARPE19 cells, a finding not previously reported.

### ADAM10, but not AXL, regulates both E- and N-cadherin protein levels

**Figure 8:**
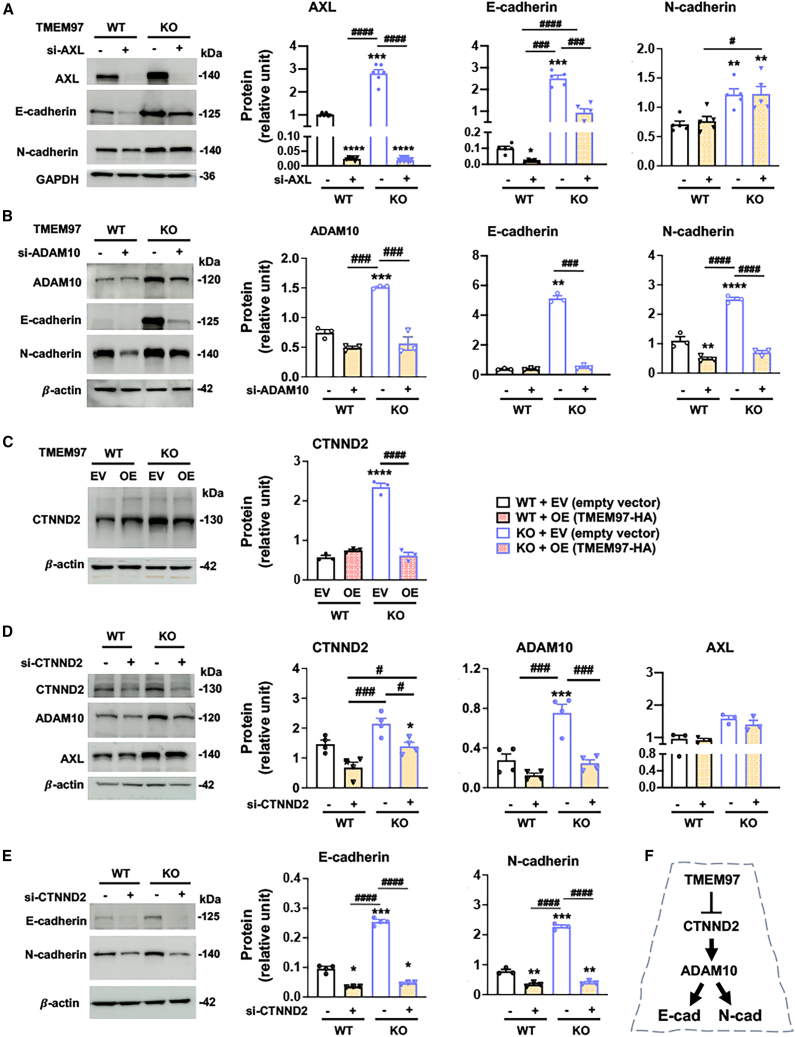
TMEM97 loss and gain of function reveal a CTNND2-ADAM10 axis that supports E- and N-cadherin protein levels To align WT and TMEM97 KO ARPE19 cells to an epithelial cell state, the cells were cultured to full confluence and then maintained for 3 days prior to harvest for immunoblot assays. For the rescue of TMEM97 expression, lentivirus for the EV control or for TMEM97 OE was used to transduce WT and KO cells that reached full confluence. The transduction was maintained for 3 days prior to harvest for immunoblotting. Quantification: mean ± SEM, *n* = 3–4 independent repeat experiments. Student’s t test (2 groups) or one-way ANOVA/Tukey (4 groups): ∗*p* < 0.05; ∗∗*p* < 0.01; ∗∗∗*p* < 0.001; ∗∗∗∗*p* < 0.0001 (compared to the first bar); ^#^*p* < 0.05; ^###^*p* < 0.001; ^####^*p* < 0.0001 (pairwise comparison). (A) AXL regulates E-cadherin but not N-cadherin protein levels. (B) ADAM10 regulates both E- and N-cadherin protein levels. (C) TMEM97 negatively regulates CTNND2 protein levels. (D) CTNND2 knockdown affects ADAM10 but not AXL protein levels. (E) CTNND2 knockdown reduces both E- and N-cadherin protein levels. (F) Schematic of the TMEM97-CTNND2-ADAM10 pathway that regulates the concurrent E- and N-cadherin expression.

We next investigated the mechanisms underlying the TMEM97 regulation of RPE cell pEMT. Using the co-expression of E- and N-cadherins as the readout of the observed pEMT state, we determined AXL, ADAM10, and POSTN, the top-ranked cadherin/adhesion-associated proteins on the heatmaps (Figure 3), as potential regulators of E- and N-cadherin protein levels. We first checked AXL because of its prominent changes not only on the protein heatmap and volcano plots but also on the mRNA heatmap. Somewhat surprising to us, silencing of AXL with small interfering RNA (siRNA) reduced E-cadherin, but not N-cadherin, in WT cells and TMEM97 KO cells (Figure 8A). Similarly, silencing of POSTN only slightly reduced E-cadherin but had no effects on N-cadherin protein levels (Figure S5). In contrast, silencing of ADAM10 abolished the TMEM97 KO-induced upregulation of both E-cadherin and N-cadherin proteins (Figure 8B). These results highlight the robust function of ADAM10 as a positive regulator of both E- and N-cadherin protein levels. This is a novel finding, especially given the lack of literature information on the functions of ADAM10 and AXL in regulating pEMT in RPE cells.

### TMEM97 negates CTNND2 protein and CTNND2 positively regulates ADAM10 protein levels

ADAM10 is only sparsely studied in RPE cells, rendering the regulation of its expression elusive. To address this, we looked into β-catenin, a component of the cadherin junction and a transcription factor whose translocation to the nucleus activates pro-EMT gene expression.^40^ However, the β-catenin protein level was not affected by TMEM97 ablation (Figure 4B). Instead, our transcriptomic analysis revealed an upregulation of CTNND2 (catenin δ-2), a lesser-known member of the catenin family. Immunoblot assays showed that TMEM97 depletion led to an increase of CTNND2 protein in ARPE19 cells, which was reversed by the rescue of TMEM97 expression in the KO background (Figure 8C). This result demonstrates a specific role for TMEM97 in negatively regulating CTNND2 protein levels, which has not been previously reported.

Given this observation, we surmised that CTNND2 might have mediated the TMEM97 regulation of the downstream ADAM10-cadherin pathway. Indeed, silencing of CTNND2 with siRNA in ARPE19 cells reduced ADAM10 but not AXL protein (Figure 8D). Furthermore, CTNND2 knockdown led to reduced E- and N-cadherin protein levels (Figure 8E). We have therefore identified a novel TMEM97-CTNND2-ADAM10 pathway that regulates the concurrent expression of E- and N-cadherin proteins in the pEMT process of ARPE19 cells (Figure 8F).

## Discussion

Our major finding is that TMEM97 governs the RPE-cell pEMT that is hallmarked by E- and N-cadherin co-expression through the CTNND2-ADAM10 axis. pEMT is a recently recognized process whose underlying regulations remain poorly understood,^5^ especially in RPE cells. TMEM97 is a drug-binding site implicated in major diseases such as cancer,^41^ yet its molecular functions remain obscure.^22^ Our study, identifying TMEM97 as a novel regulator of RPE-cell pEMT, bridges these gaps, opening new opportunities for potential interventions to mitigate RPE dysfunction in retinal diseases.

The conventional model of EMT has focused on a binary switch from E-cadherin to N-cadherin expression.^37^ However, recent research has revealed stable pEMT phenotypes. Unlike complete EMT, pEMT is characterized by the co-expression of E- and N-cadherins, along with a blend of epithelial and mesenchymal markers.^4^ pEMT is more likely to occur under mild physiological or pathological conditions, potentially leading to its oversight, in contrast to the dramatic changes in complete EMT induced by potent stimulants such as recombinant transforming growth factor-β. However, cells in a pEMT state maintain adhesive properties and often form clusters,^42^ resulting in even more aggressive tumor initiation and metastasis compared to cells undergoing full EMT.^39^^,^^43^ These insights underscore the clinical relevance of pEMT in disease progression and highlight the importance of studying this intermediate EMT state for developing effective therapeutic strategies.

The concept of pEMT evolved primarily in cancer research. Although pEMT-like states of RPE cells have been suggested, there is a lack of specific investigation into this area. For instance, in one study using ARPE19 cells, treatment with sorafenib resulted in the upregulation of both E-cadherin and N-cadherin. However, the interpretation focused on EMT without delving into the possibility of pEMT.^26^ Another report^44^ described an EMT-like phenotype in mouse RPE cells following the loss of Bbs8, yet there was insufficient data to identify whether it was a pEMT state. In addition, a study using human embryonic stem cell-derived RPE cells suggested a gradient of EMT states ranging from none to partial and complete but did not observe the simultaneous elevation of E- and N-cadherin proteins.^6^ Overall, pEMT in RPE cells remains largely unexplored.

To our knowledge, we are the first to reveal that TMEM97 functions as a molecular brake, the removal of which leads to the transition of ARPE19 cells into a pEMT state. This observation is supported by multiple lines of evidence from proteomics, transcriptomics, immunoblots, cell biology, and segmentation morphometric analysis. Our finding of TMEM97 suppressing pEMT is unexpected and unique at least in two ways. First, we initially planned to study the role of TMEM97 in RPE cell responses to oxidative stimulation; however, proteomic analysis highlighted that regardless of oxidant exposure, TMEM97 regulates cadherin/adhesion-binding pathways. Second, our finding that TMEM97 suppresses the levels of both E- and N-cadherins is distinct from the findings in cancer research. For example, TMEM97 silencing with siRNA increased E-cadherin but decreased N-cadherin and vimentin in HCT116/R and SW480/R colorectal cancer cell lines.^45^ Similarly, TMEM97 silencing in breast cancer cell lines MCF-7 and MDA-MB-231 elevated E-cadherin protein levels but reduced N-cadherin and vimentin along with other classic EMT markers such as ZEB1, Twist, Snail, and Slug.^40^ These reports using cancer cell lines support a role for TMEM97 in promoting full EMT in cancer cells. The reason for the disparity between these and our findings is not clear at present. It may stem from different cell sources and/or cell states. Whereas we started with ARPE19 cells mostly at an epithelial cell state prior to TMEM97 knockdown, the other studies used cancer cell lines, which were possibly at mesenchymal states when TMEM97 siRNAs were applied. Moreover, it is important to note that while TMEM97 has been shown to be an oncogene in colon, gastric, and breast cancers, evidence also exists for its potential tumor-suppressor role in pancreatic and prostate cancers.^18^^,^^41^^,^^46^^,^^47^ Thus, while highlighting a possible context dependence of the biological functions of TMEM97, these reports, together with our study, underscore the novelty and significance of our findings obtained in a different cell type/pathology setting. More in-depth research in this regard is needed to better understand the complexity of TMEM97-associated disease mechanisms and its potential for therapeutic targeting.

Some proteins like β-catenin, ZEB1, Snail, and Twist are well established as pro-EMT transcription factors, but the transcription factors involved in maintaining pEMT are under-studied.^5^^,^^48^ Intriguingly, our data from ARPE19 cells indicated that CTNND2 (encoding catenin δ-2), rather than the EMT driver β-catenin, was upregulated in TMEM97 KO cells undergoing pEMT. Traditionally considered a neuroprotein, CTNND2 is a lesser-studied member of the catenin family. Genetic variants of *CTNND2* have been associated with attention-deficit/hyperactivity disorder, autism, and myopia.^49^ However, its role in RPE cells remains largely unknown. To date, only one study has linked CTNND2 to the RPE, showing its higher expression in the peripheral RPE compared to the central region in rat eyes, but without exploring its influence on EMT markers.^50^ Our study here demonstrates that silencing of CTNND2 in ARPE19 cells results in reduced levels of both E-cadherin and N-cadherin proteins, providing the first evidence that CTNND2 acts as a novel regulator of pEMT in RPE cells by supporting the simultaneous expression of these cadherins. Our finding is echoed by an observation using medulloblastoma cell lines, where silencing of CTNND2 reduced E-cadherin protein, although data on N-cadherin were not available.^51^

Furthermore, our study reveals a novel CTNND2-ADAM10 axis that regulates the levels of E- and N-cadherin proteins, whereas there are no previous reports specifically linking CTNND2 to ADAM10 in any cell type. We found that CTNND2 promotes the protein level of ADAM10, which in turn supports E- and N-cadherin protein expression. ADAM10, also known as α-secretase, has been implicated in EMT in cancer cells.^52^ However, its role in EMT associated with retinal diseases remains poorly understood. One study addressed this in RPE cells, where either pharmacological inhibition or silencing of ADAM10 elevated epithelial marker proteins and reduced mesenchymal marker proteins in Epstein-Barr virus (EBV)-transformed RPE cells.^26^ Of note, both E- and N-cadherin protein levels were elevated, contradicting our finding of ADAM10 positively regulating E- and N-cadherins. The reason for this discrepancy is unclear at present; the EBV infection of ARPE19 cells is a possible factor. However, in a study using the renal carcinoma cell line A498, ADAM10 silencing resulted in the downregulation of E-cadherin protein.^52^ Although data on N-cadherin were unavailable in this report, the positive regulation of E-cadherin by ADAM10 aligns with our observation here. As a metalloprotease, ADAM10 can shed the ectodomains of various membrane proteins, including cell adhesion molecules like E- and N-cadherins, leading to their internalization and downregulation.^53^ However, ADAM10-mediated shedding/downregulation of cadherins does not explain the positive regulation by ADAM10 of E- and N-cadherins observed in our experimental setting. Indeed, we did not detect substantial E- and N-cadherin shedding based on immunoblots (Figure S6). Therefore, the precise mechanism by which ADAM10 supports E- and N-cadherin protein levels requires further investigation.

While our data indicate a role for CTNND2 in promoting the expression of both E- and N-cadherins through ADAM10, we cannot rule out the possibility that CTNND2 may also directly regulate the protein levels of these cadherins. CTNND2 belongs to the p120 (also known as catenin δ-1, encoded by *CTNND1*) subfamily.^54^ It is known that p120 binds to and stabilizes E-cadherin at junctional complexes in epithelia,^55^ and it is also found to associate with N-cadherin.^56^ Moreover, there is evidence that CTNND2 competes with p120 for binding with E-cadherin.^54^ With these facts in mind, it is an interesting question whether CTNND2 could function like p120, directly binding and stabilizing E-cadherin in RPE cells. In addition, the mechanism by which TMEM97 regulates CTNND2 expression remains to be elucidated. In this regard, potential TMEM97 interactions with chromatin-associated proteins^23^ raise a possibility that TMEM97 may influence chromatin activities—hence, gene expression.

Nevertheless, our findings uncover a novel TMEM97—| CTNND2 → ADAM10 pathway that regulates the concomitant E- and N-cadherin protein expression, a hallmark of pEMT. Given the druggability of both TMEM97/S2R and ADAM10, further elucidation of this mechanism holds promise for precision interventions aimed at protecting the RPE and retinal integrity. Considering the highly conserved sequence of the TMEM97 protein (82.4% identical) and its similar expression patterns in mice and humans,^19^ findings from mouse models on TMEM97 function are likely translatable to humans.

### Limitations of the study

Our study has limitations that warrant future investigations. While pEMT has been linked to aggressive cancer cell behaviors, its significance for the RPE and retinal degeneration remains largely unexplored. Our *in vitro* data indicated that TMEM97 ablation elevated epithelial markers E-cadherin and ZO-1, an effect that seemingly benefits the morphological stability of the RPE. However, clustering of TMEM97-deficient RPE cells may lead to disruption of the RPE cell monolayer. Consistently, our previous report^11^ suggested more severe RPE damage in TMEM97 KO mice compared to WT controls. Thus, whether TMEM97-associated pEMT promotes or mitigates RPE damage and retinal degeneration remains to be delineated. Along this line, although the rescue of TMEM97 expression in the TMEM97 KO mouse retina reduced oxidant-stimulated ROS production, our data cannot distinguish whether the alleviation of photoreceptor loss was primarily attributable to reduced ROS or suppression of pEMT in RPE cells− a possibility based on our *in vitro* data. In addition, with limited knowledge available on the role of TMEM97 in retinal degeneration, caution should be taken in future pharmacological interventions targeting TMEM97. For instance, while TMEM97 deficiency exacerbated photoreceptor loss in an oxidant-induced RPE-damage model,^11^ other studies suggested that pharmacological inhibition of TMEM97/S2R could be beneficial in different retinal degeneration models.^14^^,^^57^ Thus, to validate our findings *in vivo* and establish their relevance in broader pathophysiological contexts, further studies are required, preferably incorporating conditional TMEM97 KO, additional retinal degeneration models, and patient-derived samples.

### Conclusions

Through combined proteomics, transcriptomics, and biochemical/cellular analyses, our study bridges two under-studied domains: pEMT in RPE cells and molecular functions of TMEM97. Our research demonstrates that TMEM97 deficiency induces a pEMT phenotype in ARPE19 cells featuring simultaneous upregulation of E- and N-cadherin proteins. This effect is at least partially mediated through the regulation of the CTNND2-ADAM10 axis by TMEM97 that promotes the protein levels of both E- and N-cadherins. Further investigation into the TMEM97—| CTNND2 **→** ADAM10 pathway may lead to precise and actionable therapeutic strategies to treat retinal degeneration.

## Materials and methods

### Materials

Unless otherwise noted, the majority of the reagents used in this study were procured from Thermo-Fisher Scientific, and the detailed information can be found in Table S1. All chemical compounds were sourced from Sigma-Aldrich (St. Louis, MO) or as otherwise specified.

### Animals

All animal procedures were approved by the Institutional Animal Care and Use Committee and were performed in accordance with the Association for Research in Vision and Ophthalmology statement for the use of animals in ophthalmic and vision research. The mice used in the study were kept on a 4% fat diet (Harland Teklad, 8604 M/R) and maintained under standard light/dark cycles (12 h/12 h). Male mice aged 40–50 days were chosen for the experiment.

### *Tmem97*^−/−^ (KO) mice

The TMEM97 KO mouse line was established as previously described.^11^ Cryopreserved sperm from the C57BL/6N-Tmem97tm1.1(KOMP)Vlcg strain was acquired from the KOMP Repository at University of California, Davis (stock no. 10753A-D5). This strain was revived on the C57BL/6J background (JAX no. 000664). Homozygous KO mice were used in the experiments. The primers for genotyping are the following: WT (forward) (5′-GGGTAACATTTGAATTATGGCTAG-3′) and WT (reverse) (5′-CACACTGGGGGCTCCTGCATC-3′); KO (forward) (5′-ACTTGCTTTAAAAAACCTCCCACA-3′) and KO (reverse) (5′-GGTGTCACACACCTTTAATCCCAGC-3′).

### Subretinal lentivirus delivery for the rescue of TMEM97 expression in TMEM97 KO mice

TMEM97 KO mice were anesthetized with a combination of ketamine (100 mg/kg) and xylazine (10 mg/kg).^58^ To maximize pupil dilation for subretinal injection, both tropicamide (1%) and phenylephrine (2.5%) eye drops were applied.^58^ This combination has been used in preclinical^59^ and clinical studies.^60^ Once anesthetization was confirmed, mice were placed on a heated pad to maintain body temperature and positioned under a surgical microscope with an eye exposed. Subretinal injection was performed as previously described.^61^ Briefly, sclerotomy was made with a 30G beveled needle in the sclera 1 mm posterior to the limbus, and a blunt 35G needle attached to a glass micro-syringe loaded with lentivirus was gently advanced through the sclerotomy into the subretinal space. The lentivirus (1 μL, >10^9^ infectious units/mL) was slowly delivered, creating a localized bleb of fluid. The needle was carefully withdrawn, while gentle pressure was applied to the injection site to minimize leakage. Erythromycin ointment was applied to the ocular surface to prevent postsurgical infection. After recovery on the heated pad, mice were returned to the housing facility.

### Oxidant-induced RPE-damage model of retinal degeneration

While there is no perfect murine model for mimicking human AMD, the oxidant (NaIO_3_)-induced model is widely acknowledged for inducing damage to the RPE and subsequent loss of photoreceptors.^1^^,^^62^^,^^63^ As we previously described,^11^ mice received a single tail-vein injection of NaIO_3_ (30 mg/kg of body weight, from Sigma-Aldrich).^64^ Injection was carried out under isoflurane anesthesia via inhalation, with a flow rate of 2 mL/min. Three days after injection, animals were euthanized in a chamber gradually filled with CO_2_.

### Spectral domain-OCT

Prior to euthanasia and 3 days after injection of NaIO_3_, mice received intraperitoneal injection of ketamine-xylazine (90 mg/kg ketamine mixed with 10 mg/kg xylazine) for anesthesia. Topical proparacaine eye drops (0.5%) were administered to further numb the eyes. To enable live animal imaging, tropicamide drops (1%) and phenylephrine (2.5%) were applied to dilate pupils. After anesthesia was achieved, topical 1% carboxymethylcellulose (Refresh Liquigel) was applied to the corneal surface. Throughout the procedure, the mice were securely positioned on a platform and maintained on a heated pad until they fully regained consciousness.

Spectral domain (SD)-OCT imaging was performed using image-guided tomography (Micron IV OCT2; Phoenix Research Laboratories). Imaging procedures were controlled using the Reveal OCT software and image analysis was conducted using ImageJ software. Single B-scans, 1.6 mm, were conducted for each eye, with an average of 100 images, each of which encompassed the optic nerve, injection site, and injected area.

Using ImageJ, outer retinal thickness was measured between the top of the outer plexiform layer reflection and the bottom of the RPE reflection at a distance of 500 μm from the optic nerve in all OCT images. There was not any removal of outlier data as we did not find any data entry mistake or measurement error.

### Immunofluorescence and confocal microscopy

We followed the protocol outlined in our previous report.^23^ Briefly, enucleated eyes were fixed overnight in 4% paraformaldehyde and then cryoprotected using 30% sucrose, from which 10-μm-thick cryosections were obtained. These sections underwent permeabilization with 0.1% Triton X-100 in PBS for 20 min and blocking with 5% normal donkey serum (017-000-121, Jackson ImmunoResearch Laboratories) for 1 h at room temperature. The sections were then incubated overnight at 4°C with an antibody for ZO-1. To visualize the specific staining, a fluorescently labeled secondary antibody (Alexa 488 conjugated donkey anti-rabbit or Alexa 594-conjugated donkey anti-mouse) was applied to the sections and incubated for 2 h at room temperature. After rinsing, the sections were counterstained with DAPI. Detailed information about the antibodies is provided in Table S2. Images were captured using a Zeiss LSM 880 with Airyscan FAST Confocal Microscope Immunofluorescence from both central and mid-peripheral regions was quantified using ImageJ.

### ARPE19 cell culture

The ARPE19 human RPE cell line was from the American Type Culture Collection (catalog no. CRL2302). These cells were cultured in DMEM/F12 medium (catalog no. 11320082, Thermo Fisher Scientific) supplemented with 10% fetal bovine serum (FBS) and penicillin-streptomycin (catalog no. 5140163, Thermo Fisher Scientific). The culture was maintained at 37°C in a humidified atmosphere with 5% CO_2_. To induce oxidative stress, a concentration of 5 mM NaIO_3_ was selected, which has been previously established in our research to elevate ROS without causing extensive cell death.^11^

### TMEM97 KO single-clone human ARPE19 cell line

The *TMEM97*^*−/−*^ (KO) single-clone cell line was generated following the CRISPR-Cas9 genome-editing methodology described in our recent report.^11^ We selected one highly effective single-guide RNA (sgRNA) sequence (5′-TCCGGCAACCAGGCGCTGCG-3′) from a pool of three candidates (Table S3) and cloned it into LentiCRISPR version 2. Lentivirus was packaged in HEK293FT cells (Invitrogen) employing pSPAX2 (Addgene, catalog no. 12260) and pMD2.G (Addgene, catalog no. 12259). ARPE19 cells were transduced with the lentivirus in DMEM/F12 supplemented with 10% FBS. Following a 3-day incubation, cells were treated with 5 μg/mL puromycin for a duration of 2 weeks. Single clones were isolated through serial dilutions. For TMEM97 OE, we cloned the cDNA of human TMEM97 open reading frame from APRE19 cells and subcloned it into the pLenti-puro vector (Addgene, catalog no. 39481) in fusion with the hemagglutinin (HA) tag at the C terminus, as recently reported.^23^ The lentivector was used to transduce APRE19 cells prior to various assays.

Throughout this study, KO cells refer to the TMEM97^−/−^ human ARPE19 cell line generated using the CRISPR-Cas9 approach through lentiviral transduction, and WT (TMEM97^+/+^) is the control cell line generated with the lentivector that expresses only the sgRNA without Cas9.

OE results are from APRE19 transduction using the lentivector to express the TMEM97-HA fusion protein unless otherwise specified in the figure legends.

### Illumination of ROS *in vitro* and *in vivo*

We used the fluorescent dye 2′,7′-dichlorodihydrofluorescein diacetate (H2DCFDA, Sigma-Aldrich, catalog no. D6883), which is permeable to cells and reactive to ROS. Upon cellular uptake, intracellular esterases cleave the acetyl groups, yielding H2DCF, which is subsequently oxidized by ROS, leading to the formation of the highly fluorescent molecule DCF46.^65^

ARPE19 cells were cultured to ∼50% confluence in the regular growth medium and then incubated without (0 h) or with 5 mM NaIO_3_ for 3, 6, or 9 h. For the rescue of TMEM97 expression, lentivirus for the EV or for the expression of TMEM97-HA was used to transduce KO cells for 3 days. The transduced cells were then incubated without (0 h) or with 5 mM NaIO_3_ for 3, 6, or 9 h. At the end of NaIO_3_ treatment, cells were transitioned to a NaIO_3_-free fresh medium and exposed to 10 μM H2DCFDA for 60 min at 37°C prior to imaging.^11^ Green fluorescence (excitation wavelength/emission wavelength: 485/528 nm) was recorded using an EVOS microscope (Thermo Fisher Scientific).

For ROS imaging *in vivo,* non-fixed retinal cryosections were incubated with 10 μM H2DCFDA for 60 min at 37°C and imaged under the EVOS microscope.

### Proteomics through mass spectrometry

*TMEM97*^*−/−*^ and *TMEM97*^*+/+*^ human ARPE19 cells were cultured to 80% confluence, treated without or with 5 mM NaIO_3_ for 24 h, and then flush-frozen in liquid N_2_ before submitting the samples to the W.M. Keck Biomedical Mass Spectrometry Laboratory at the University of Virginia. The cells were extracted using a Bead Beater. The sample was reduced with 10 mM DTT in 0.1 M ammonium bicarbonate followed by alkylation with 50 mM iodoacetamide in 0.1 M ammonium bicarbonate (both room temperature for 0.5 h). The sample (5 μg equiv) was then digested overnight at 37°C with 0.1 μg trypsin in 50 mM ammonium bicarbonate. The sample was acidified with acetic acid to stop digestion and then purified using magnetic beads and C18 tips. This extract was evaporated to 20 μL for mass spectrometry (MS) analysis. The liquid chromatography-MS system consisted of a Thermo Orbitrap Exploris 480 MS system with an Easy Spray ion source connected to a Thermo 75 μm × 15 cm C18 Easy Spray column. Around 1 μg of the extract was injected and the peptides eluted from the column by an acetonitrile/0.1 M formic acid gradient at a flow rate of 0.3 μL/min over 2.0 h. The nanospray ion source was operated at 1.9 kV. The digest was analyzed using the rapid switching capability of the instrument acquiring a full-scan mass spectrum to determine peptide molecular weights followed by product ion spectra (top 10 high-energy collisional dissociation) to determine the amino acid sequence in sequential scans. This mode of analysis produces approximately 25,000 MS/MS spectra of ions ranging in abundance over several orders of magnitude. Not all MS/MS spectra are derived from peptides.

The data were analyzed by database searching using the Sequest search algorithm against Uniprot Human. The analysis yielded about 100 proteins with significant level changes. To detect differentially abundant proteins, spectral counting values of each protein were compared among samples (*n* = 3). For statistical analysis, data were used as input for the limma package using R,^66^ and proteins with adjusted *p* (*p*adj) < 0.05 were considered as differentially abundant comparing KO and WT cell samples with or without 5 mM NaIO_3_ treatment. Volcano plots were obtained using ggplot2 in R.

### Bulk RNA sequencing and transcriptomic analysis

The experiment was conducted as we recently reported.^11^ *TMEM97*^*+/+*^ and *TMEM97*^*−/−*^ ARPE19 cells were cultured in standard growth medium (until an approximately 80% confluence) followed by RNA extraction. Triplicate cell cultures in each treatment group were used for RNA sequencing. The “splice aware” aligner, STAR,^67^ was employed to map the reads to both the transcriptome and genome, while HTseq software^68^ was used for counting aligned reads corresponding to each gene. The DESeq2 package^69^ was used for the analysis of differentially expressed genes, with genes ranked based on their log2 fold change and false discovery rate-corrected *p* values (or *p*adj). Enriched pathways were determined based on enrichment scores and normalized enrichment scores. GO terms with a *p*adj < 0.05 were deemed significantly enriched by differentially expressed genes.

### Cell morphometric analysis through segmentation

To capture the cell morphometric difference between *TMEM97*^*+/+*^ and *TMEM97*^*−/−*^ ARPE19 cells, cells were cultured to low confluence (∼50%) on coverslips and then stained with Invitrogen Molecular Probes CellTracker Red CMTPX Dye (Thermo Fisher Scientific, catalog no. C34552). Glass slides were prepared with VECTASHIELD Antifade Mounting Medium (VECTOR, catalog no. H-1200-10). Cell images were captured using a Leica Stellaris 5 confocal microscope. Individual cells were segmented by marker-controlled watershed segmentation, and cellular morphology was analyzed using MorphoLibJ in ImageJ. Circularity, defined as 4π × area/perimeter2, ranges from 0 to 1, with 1 representing a perfect circle.

### Immunoblotting

The assay was conducted following our previously reported protocol.^23^ In brief, total protein concentrations were determined using the DC Protein Assay Kit (Bio-Rad, catalog no. 5000111), with 50 μg protein loaded per sample for SDS-PAGE. Following transfer onto polyvinylidene fluoride membranes, each specific protein was detected using primary and secondary antibodies (refer to Table S2). Western blot images were captured using the Amersham Imager 680 (GE Healthcare) and analyzed using ImageJ. Protein band densitometry was initially normalized to a loading control (glyceraldehyde 3-phosphate dehydrogenase or β-actin), then to the basal condition in each experiment (as indicated by the first bar in the figures), and finally quantified as fold change. Fold changes from a minimum of three independent experiments were averaged, and mean ± standard error of the mean (SEM) was calculated.

### Quantitative reverse-transcription PCR

Cell lysates were processed for total RNA extraction using TRIzol (Thermo Fisher Scientific, catalog no. 15596026), according to the manufacturer’s protocol. Purified mRNA (1 μg) served as the template for the first-strand cDNA synthesis, and quantitative reverse-transcription polymerase chain reaction (qRT-PCR) was conducted with QuantStudio3 Real-Time PCR System (Applied Biosystems). Each cDNA template underwent amplification employing SYBR Green PCR Master Mix. The primer sequences are detailed in Table S4.

### Statistical analysis

Statistical analysis was performed using GraphPad Prism version 8.02. Before analysis, datasets underwent normality testing via the Shapiro-Wilk test. Student’s t test was employed for comparisons between two groups, while for multiple group comparisons, one-way analysis of variance (ANOVA), followed by a post hoc test (as specified in the figure legends), were conducted. Results are expressed as mean ± SEM, with statistical significance denoted as *p* < 0.05.

## Data and code availability

The data that support the findings of this study are available from the corresponding authors upon reasonable request.

## Acknowledgments

This work was supported by the NIH awards EY029809 (to L.-W.G.), R01EY028027, R01EY031039, R01AG082108, and R01EY032512 (to B.D.G.), the UVA Strategic Investment Fund and 10.13039/100000002NIH grant R01EY029799, the DuPont Guerry, III Professorship, and a gift from Mr. and Mrs. Eli W. Tullis (to J.A.).

## Author contributions

Conceptualization, B.D.G. and L.-W.G. Methodology, J.L., Y.N., H.S., S.D., and N.S. Data curation, J.L., Y.N., H.S., and X.Z. Formal analysis, J.L., Y.N., X.Z., and S.D. Investigation, J.L., Y.N., and L.-W.G. Visualization, X.Z. Writing – original draft, J.L. and L.-W.G. Writing – review & editing, J.M., J.A., B.D.G., and L.-W.G. Supervision, J.M., J.A., B.D.G., and L.-W.G. Project administration, L.-W.G. Funding acquisition, J.A., B.D.G., and L.-W.G.

## Declaration of interests

J.A. is a co-founder of iVeena Holdings, iVeena Delivery Systems, and Inflammasome Therapeutics; a consultant for Retinal Solutions and Saksin LifeSciences; and a board member of Theragen Biologics.

## Supplemental information

Document S1. Figures S1–S6 and Tables S1–S4

Document S2. Article plus supplemental information
