# Supplementary material for: TMEM97 governs partial epithelial-mesenchymal transition of retinal pigment epithelial cells via the CTNND2-ADAM10 axis
Source: Mol Ther Nucleic Acids. 2025 Jan 21;36(1):102460. doi: 10.1016/j.omtn.2025.102460 (PMC11848774; doi:10.1016/j.omtn.2025.102460)
Supplement: Document S1. Figures S1–S6 and Tables S1–S4 [file mmc1.pdf]

OMTN, Volume *36*

Supplemental information

TMEM97 governs partial epithelial-mesenchymal transition of retinal pigment epithelial cells via the CTNND2-ADAM10 axis

Jing Li, Yosuke Nagasaka, Hongtao Shen, Xinyu Zhou, Jianjie Ma, Dilza Trevisan-Silva, Nicholas E. Sherman, Jayakrishna Ambati, Bradley D. Gelfand, and Lian-Wang Guo

Supplemental figures

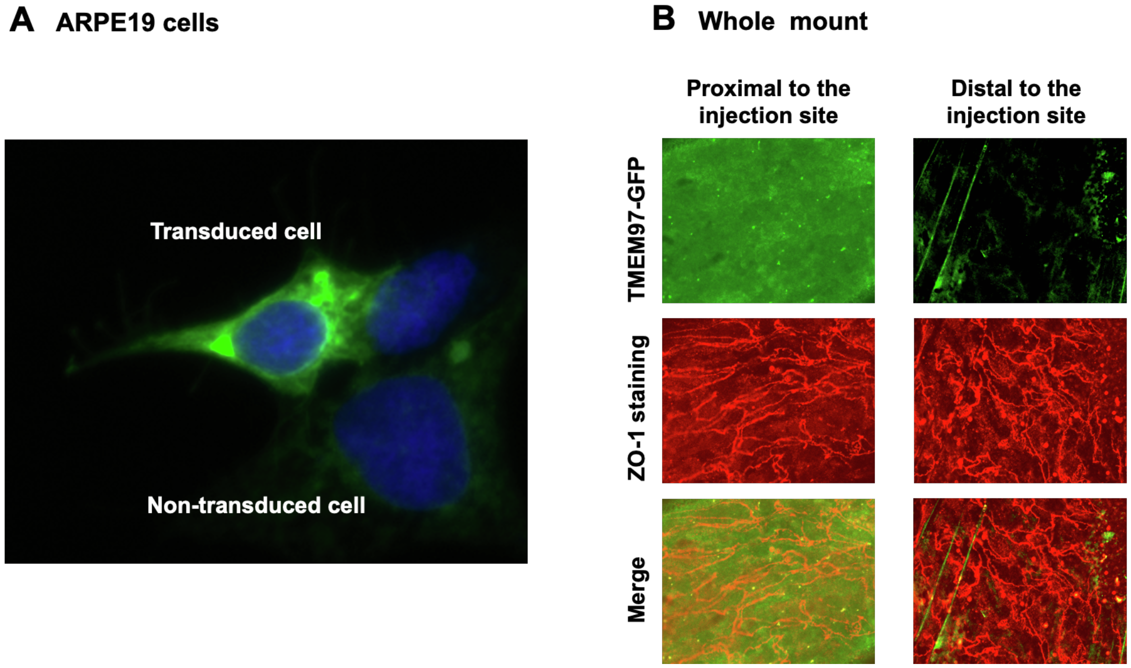

Figure S1. TMEM97-GFP expression in ARPE19 cells and in the RPE of *Tmem97-/-* mice.

A. *Expression in ARPE19 cells. Lentivirus was used to transduce ARPE19 cells to express TMEM97-GFP. Cells were fixed and DAPI-stained prior to microscopy.*
B. *Expression in the RPE. Lentivirus for TMEM97-GFP expression was subretinally delivered into male Tmem97-/- mice and allowed to express for 3 weeks. The mice then received a single tail-vein injection of $NaIO_3$ (30 mg/kg). Three days later, the mice were euthanized. Eye cups were prepared and immunostaining of ZO-1 and GFP was performed on whole mounts.*

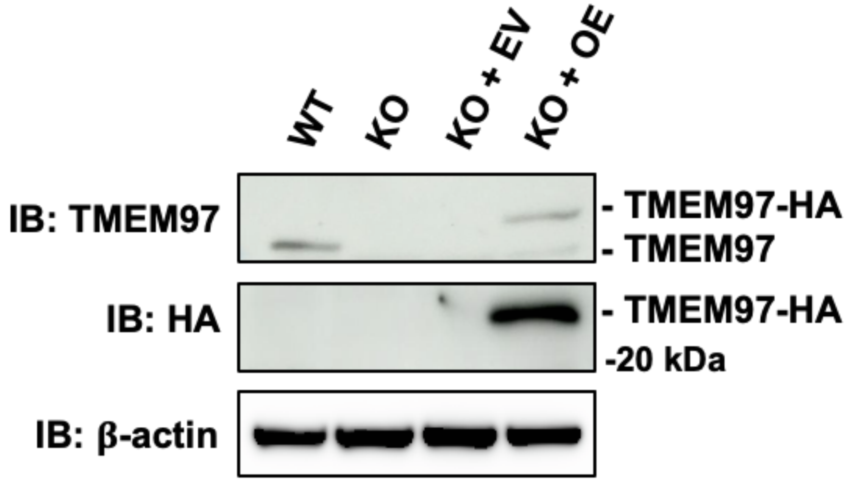

Figure S2. Rescue of TMEM97 expression in TMEM97-KO ARPE19 cells

Cells were cultured to full confluence and maintained for another 3 days prior to harvest for immunoblot assays. For the rescue of TMEM97 expression, lentivirus for the empty vector (EV) or for the expression of TMEM97-HA (OE) was used to transduce WT and KO cells that reached full confluence, and the culture continued for 3 days prior to harvest for immunoblotting.

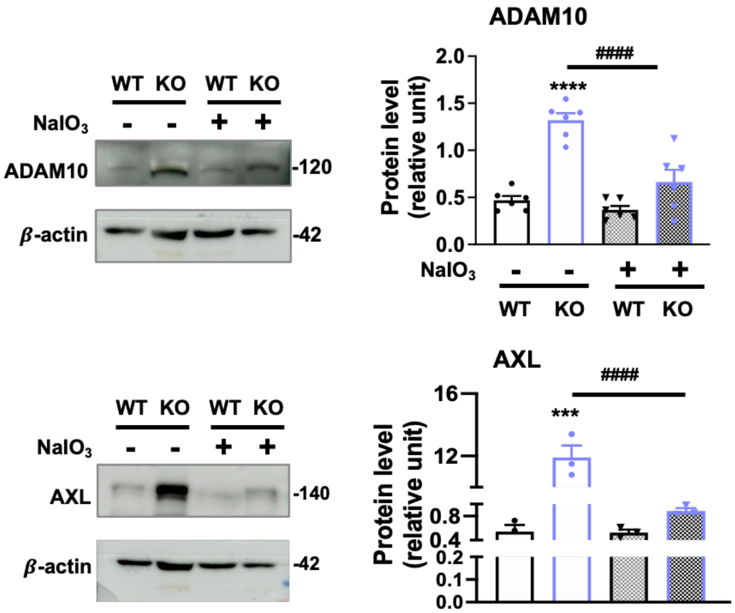

Figure S3. Upregulation of ADAM10 and AXL proteins in TMEM97-/- ARPE19 cells in the absence or presence of NaIO₃.

*To align WT and TMEM97-KO ARPE19 cells to an epithelial cell state, the cells were cultured to full confluence and then maintained for 2 days prior to treatment without or with 5mM $NaIO_3$ for 24h. The cells were then harvested for immunoblot assays. Quantification: Mean ± SEM, n= 3-4 independent repeat experiments. One-Way ANOVA/Tukey: ***P<0.001, ****P<0.0001 (compared to the first bar); ####P<0.0001 (pair-wise comparison).*

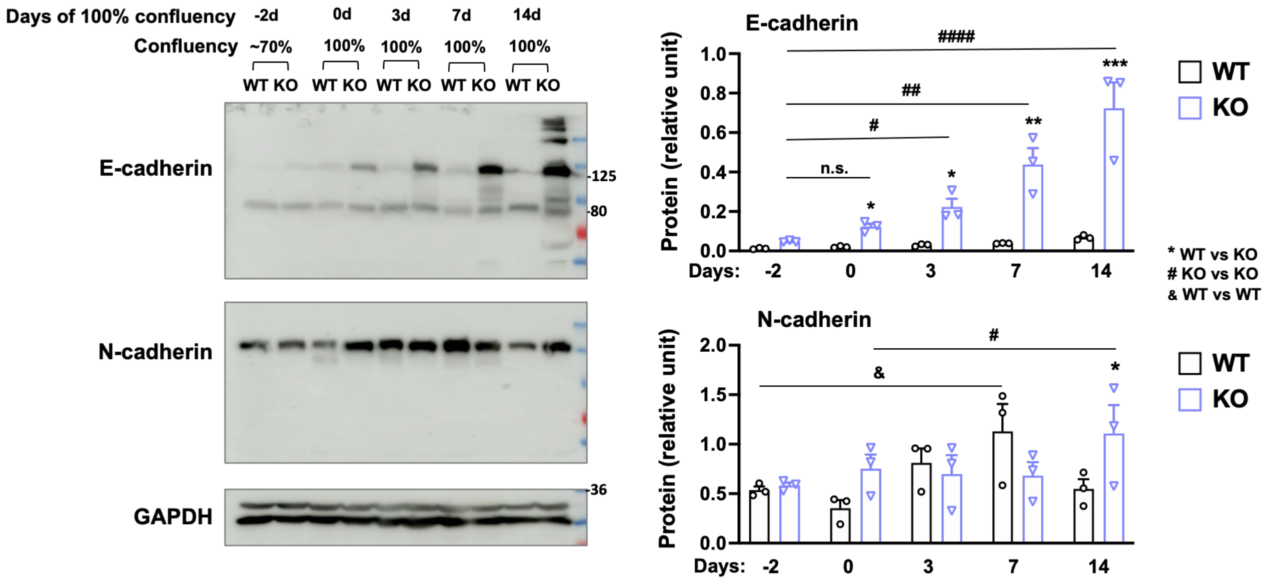

Figure S4. Time course of upregulation of E-cadherin and N-cadherin proteins in TMEM97-/- ARPE19 cells.

*Cultured WT and TMEM97-KO ARPE19 cells were harvested for immunoblot assay at different time points: 70% confluence (2 days before full confluence), 100% confluence (0 day after full confluence), and 3, 7, and 14 days after reaching 100% confluence. Quantification: Mean ± SEM, n= 3-4 independent repeat experiments. Two-way ANOVA/Tukey: *P<0.05, P<0.01, P<0.001 (KO vs WT); #P<0.05, ##P<0.01, ####P<0.0001 (KO vs KO); &P<0.05 (WT vs WT).*

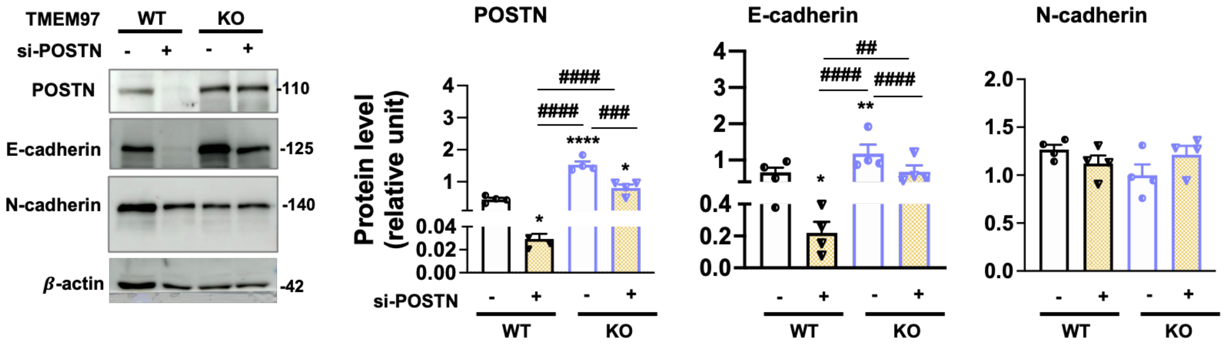

Figure S5. Lack of effect of POSTN knockdown on E- and N-cadherin protein levels.

*To align WT and TMEM97-KO ARPE19 cells to an epithelial cell state, the cells were cultured to full confluence and then maintained for 3 days prior to harvest for immunoblot assays. siRNA was added to the culture on day 0 of 100% confluence and the transfection continued for 3 days. Quantification: Mean ± SEM, n= 3-4 independent repeat experiments. One-way ANOVA/Tukey analysis: *P<0.05, **P<0.01, ****P<0.0001 (compared to the first bar); ##P<0.01, ###P<0.001, ####P<0.0001 (pair-wise comparison).*

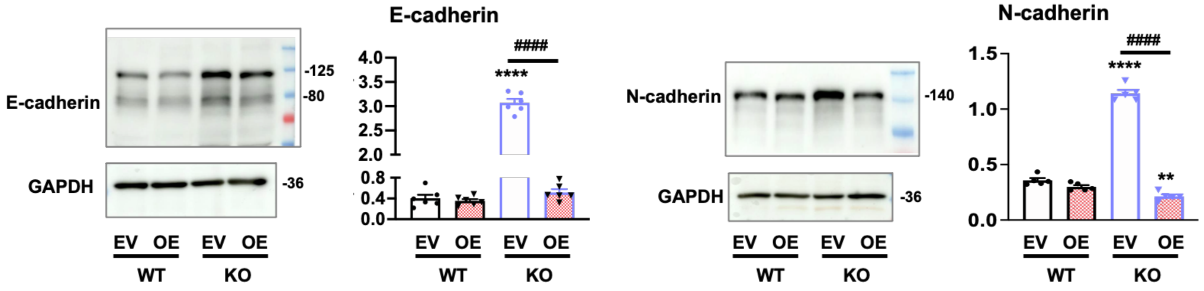

Figure S6. Immunoblots show no substantial cleavage of E-cadherin and N-cadherin

*To align WT and TMEM97-KO ARPE19 cells to an epithelial cell state, the cells were cultured to full confluence and then maintained for 3 days prior to immunoblotting. For the rescue of TMEM97 expression, lentivirus for the pLenti-HA (EV) or for the expression of TMEM97-HA (OE) was used to transduce WT and KO cells on day-0 of full confluence. The culture continued for 3 days prior to cell harvest. Quantification: Mean ± SEM, n= 3-4 independent repeat experiments. Statistics: One-way ANOVA/Tukey, **P<0.01, ****P<0.0001 (compared to the first bar, EV/WT); ####P<0.0001 (pair-wise comparison).*

Supplemental tables

Table S1. Major reagents and materials

Name	Company	Catalog number
Sodium iodate	Sigma-Aldrich	S4007
H2DCFDA	Sigma-Aldrich	D6883
Calcein AM, cell-permeant green dye	Invitrogen	C34852
jetPRIME® transfection reagent	Polyplus	101000046
Lipofectamine RNAiMAX transfection reagent	Invitrogen	13778150
RIPA lysis buffer	Invitrogen	89901
Hu ADAM10 siRNA1	Invitrogen	4427038 Assay ID s1005
Hu ADAM10 siRNA2	Invitrogen	4427038 Assay ID s1004
Hu POSTN siRNA	Invitrogen	4392420, Assay ID: 20887
Hu AXL siRNA	Invitrogen	4390824, Assay ID s1845
Hu CTNND2 siRNA1	Invitrogen	4427037, S3728
Hu CTNND2 siRNA2	Invitrogen	4427037, s3730

Table S2. Antibodies used for western blotting (WB) and immunofluorescence (IF)

Antibody	Company	Catalog number	Dilution
ADAM10	Proteintech	66620-1-Ig	1:1000(WB)
CTNND2	Invitrogen	MA5-47114	1:1000(WB)
POSTN	Proteintech	66491-1-Ig	1:1000(WB)
AXL	Proteintech	13196-1-AP	1:1000(WB)
E-cadherin	Proteintech	20874-1-AP	1:1000(WB), 1:100(IF)
N-cadherin	Cell signaling technologies	13116	1:1000(WB), 1:100(IF)
TMEM97	Proteintech	26444-1-AP	1:1000(WB)
ZO-1	Invitrogen	33-9100	1:1000(WB)
CyclinD1	Cell signaling technologies	55506	1:1000(WB)
β-catenin	Invitrogen	13-8400	1:1000(WB)
α-SMA	Proteintech	14395-1-AP	1:1000(WB)
Fibronectin	Proteintech	15613-1-AP	1:1000(WB)
Vimentin	Proteintech	22031-1-AP	1:1000(WB)
β-actin	Proteintech	21327-1-AP	1:5000(WB)
GAPDH	Cell Signaling technologies	2118S	1:3000(WB)

Table S3. Candidate sgRNAs for CRISPR/Cas9-mediated TMEM97 KO

	sense	antisense
1	5'-caccgTCGACTGGGTAGAGCTCGCG	5'-aaacCGCGAGCTCTACCCAGTCGAc
2	5'-caccgGCGCGAGCTCTACCCAGTCG	5'-aaacCGACTGGGTAGAGCTCGCGCc
3	5'-caccgTCCGGCAACCAGGCGCTGCG	5'-aaacCGCAGCGCCTGGTTGCCGGAc

Note: Number 3 was chosen for the KO experiment and selection of single clones.

Table S4. Sequence of primers for qRT-PCR

Name	Forward	Reverse
Ki67	TCTGGGTTACCTGGTCTTAGTT	GCGTATTAGGAGGCAAGTTTT
PCNA	GAAGCACCAAACCAGGAG	CCAGAAGGCATCTTTACTACAC
